# Real‐time mildew detection and gradation in simulated containerized soybeans: Insights from GC‐IMS analysis of mVOCs and VOCs

**DOI:** 10.1002/fsn3.4302

**Published:** 2024-07-09

**Authors:** Xuejian Song, Lili Qian, Lixue Fu, Rongan Cao, Xinhui Wang, Mingming Chen

**Affiliations:** ^1^ College of Food Science Heilongjiang Bayi Agricultural University Daqing China; ^2^ Key Laboratory of Agro‐Products Processing and Quality Safety of Heilongjiang Province Daqing China; ^3^ National Coarse Cereals Engineering Research Center Daqing China

**Keywords:** bulk grain, GC‐IMS, mildew, soybean, volatile organic compounds

## Abstract

In the context of bulk grain container transportation, the complex logistics can lead to grain mildew and subsequent economic losses. Therefore, there is a pressing need to explore swift and real‐time mildew detection technology. Our investigation, simulating actual transportation conditions, revealed that *Aspergillus*, *Penicillium*, and *Rhizopus* were the primary molds responsible for soybean mildew during container transportation. Utilizing gas chromatography‐ion migration spectroscopy (GC‐IMS), we analyzed the correlation between the mVOCs (microbial volatile organic compounds) produced by dominant mold and the VOCs emitted during soybean mildew. Principal Component Analysis (PCA) and clustering results demonstrated the distinctive identification of VOCs in soybeans with varying degrees of mildew. The mildew degree significantly influenced the content variation of VOCs. As the mildew degree increased, the concentrations of nonanal, octanal, etc. progressively decreased, contrasting with the rising levels of phenylacetaldehyde, 3‐methyl‐2‐butenal, etc. Therefore, the combination of GC‐IMS with chemometrics proves to be a viable method for identifying the mildew degree of soybeans. Therefore, this study underscores the importance of implementing effective mildew detection techniques in the challenging context of bulk grain container transportation.

## Graphical abstract

Utilizing gas chromatography‐ion migration spectroscopy (GC‐IMS), we analyzed the correlation between the VOCs (volatile organic compounds) produced by dominant mold and the VOCs emitted during soybean mildew. Principal Component Analysis and clustering results demonstrated that as the mildew degree increased, the concentrations of nonanal, octanal, etc. progressively decreased, contrasting with the rising levels of phenylacetaldehyde, 3‐methyl‐2‐butenal, etc. Therefore, the combination of GC‐IMS with chemometrics proves to be a viable method for identifying the mildew degree of soybeans.

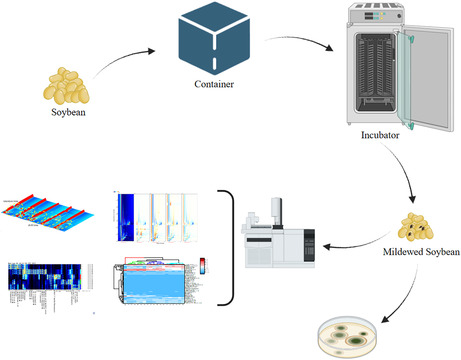

## 1 INTRODUCTION

Soybean (*Glycine max* (L.) Merr.), an annual herb in the Leguminosae genus, serves as a crucial nutrient and a primary source of plant protein and oil (Rizzo & Baroni, 2018). Comprising approximately ~40% protein (dry weight) with a favorable amino acid composition, it includes glutamic acid, arginine, leucine, aspartic acid, etc. It benefits human health. Additionally, it contains 20% cholesterol‐free oil (dry weight), predominantly rich in unsaturated fatty acids, along with 33% carbohydrates and minerals (Zhang et al., 2020). Additionally, soybeans harbor various bioactive substances, including oligosaccharides, saponins, isoflavones, phytosterols, trypsin inhibitors, and bioactive peptides (Pan et al., 2018; Zhang et al., 2017; Zhu et al., 2018). Beyond its role as an oil and food crop, soybean is both an industrial raw material and a cash crop. China stands not only as a significant soybean producer but also as a major importer (Lv et al., 2022). To maintain a stable soybean market supply, a substantial quantity of soybeans necessitates annual cross‐regional transportation. Container multimodal transport has emerged as the predominant method due to its cost‐effectiveness, minimal loss, high‐volume capacity, and rapid speed (Liu, Bai, & Chen, 2017; Liu, Deng, et al., 2017; Meng, 2020). However, the unpredictable transportation time, influenced by complex environmental conditions, poses challenges, leading to the potential risk of soybean mold in containers (Li et al., 2024). Soybean mold is characterized by the formation of gray‐green to black mold spots on the surface of soybeans, which not only reduce the commercial value of soybeans but may also produce toxins such as aflatoxin, which pose a threat to human health. Moldy soybeans are prone to breakage and further decay during transport, increasing transport losses, and may also pose a risk to consumer health due to the presence of toxins. At the same time, the moisture content of soybeans is also the main cause of mold and elevated temperatures in the storage environment. The higher the moisture content of transported soybeans, the faster the respiration, resulting in higher ambient temperatures and thus a greater probability of mold. Different molds may produce the same mVOCs. Therefore, early detection of soybean mold contamination, prompt implementation of control measures, and mitigation of economic losses become imperative.

Traditional methods for detecting mold contamination in cereals include culture (Brodsky et al., 1982), chromatography (Gonzalez Pereyra et al., 2011), immunological analysis (ELISA) (Yu et al., 2011), and polymerase chain reaction (PCR) (Mateo et al., 2011). However, traditional culture detection methods suffer from prolonged detection times, resulting in poor detection results. PCR technology may yield false negatives when the detected microbial DNA inhibits enzyme activity. Chromatography operations are complex, requiring multiple elutions, and ELISA test kits are non‐reusable, contributing to high testing costs. Therefore, as a response to these challenges, researchers have explored a non‐destructive technology for grain mold contamination detection that is simple, efficient, and accurate. Imaging technology, operating within a limited electromagnetic range, provides information about grain grains. Studies indicate that imaging technology achieves a correct discrimination rate of 94.3% (Singh et al., 2022). Furthermore, analysis using linear discriminant analysis (LDA) and quantitative description analysis (QDA) enhances discrimination accuracy to 97% (Chelladurai et al., 2010). Compared to surface‐based imaging, spectral technology, focusing on internal structure analysis, is more effective in distinguishing grain mold contamination. Shen et al. (2018) combined Near Infrared (NIR) with computer vision to online detect and distinguish corn grains contaminated by mold, achieving a remarkable 100% accuracy. Mahlein et al. (2019) utilized Hyperspectral Imaging (HSI) technology to monitor *Fusarium* contamination in wheat grains, revealing significantly higher spectral reflectance in contaminated grains compared to healthy ones, thus demonstrating the effectiveness of HSI in effectively monitoring *Fusarium* contamination in wheat. While both imaging and spectroscopy technologies are apt for distinguishing moldy grains from healthy ones, their efficacy improves with higher mold contamination (Baek et al., 2020; Zareef et al., 2021). However, employing these technologies for detection requires substantial time for dataset processing (Fox & Manley, 2014), making it challenging to extract characteristic spectral image information and establish a discriminant model.

Gas Chromatography‐Ion Migration Spectrometry (GC‐IMS), a novel detection technology, offers excellent resolution for isomers and substances with similar polarities, enhancing the accuracy of detecting mixtures without the need for sample pretreatment. This capability meets the demands of rapid field analysis. The combination of gas chromatography (GC) and IMS has seen significant advancements. Before entering the IMS system, GC utilizes capillary columns to separate complex compounds into single components, thereby reducing competitive ionization and enabling the quantification of a broad concentration range of volatile and semi‐volatile compounds (Vautz et al., 2018). Additionally, combining IMS (drift time) with gas chromatography pre‐separation (retention time) enhanced selectivity, effectively achieving two‐dimensional separation of isomers and co‐eluted compounds (García‐Nicolás et al., 2020). Research indicates that GC‐IMS effectively characterizes volatile substances in various samples, making it suitable for detecting aldehydes, alcohols, ketones, esters, and aromatic compounds (Capitain & Weller, 2021). Its applications extend to grain authenticity evaluation (He et al., 2021), quality analysis (Ma et al., 2022), and, more recently, the detection of grain mold pollution. Gu, Chen, et al. (2020), Gu, Wang, et al. (2020) utilized GC‐IMS to detect mold contamination in wheat samples, concurrently verifying the infection rate of *Aspergillus flavus* in simulated field samples. The method possesses high sensitivity, a low detection limit, excellent separation efficacy, and eliminates the need for sample pretreatment. The GA‐SVM model demonstrated a robust 86.67% classification accuracy for *Aspergillus flavus* infection rates in wheat during the 0–4‐day simulation field. In another study, a GC‐IMS method was employed to detect VOCs in fungal‐contaminated rice (Gu, Chen, et al., 2021; Gu, Wang, & Wang, 2021), identifying 24 characteristic volatile substances in moldy rice. The analysis of variance‐partial least squares regression (APLSR) showed a significant correlation between target compounds and the total mold count. The established Logistic model efficiently monitors the growth of individual molds. The results highlight the applicability of GC‐IMS technology, coupled with a suitable pattern recognition algorithm, for early mold detection in rice. Gu, Chen, et al. (2021), Gu, Wang, and Wang (2021) used GC‐IMS, along with orthogonal partial least squares discriminant analysis (OPLS‐DA), to swiftly identify potential *Aspergillus flavus* contamination in peanut kernels, thus achieving an impressive 96.7% correct discrimination rate between healthy and moldy peanuts. Utilizing independent and fusion signals from PLSR, a regression model predicting the total *Aspergillus flavus* count in peanuts was established. This model effectively monitors *Aspergillus flavus* presence, offering the potential for early aflatoxin warning in peanuts.

The study investigated soybean mildew occurrence and the factors influencing it in containers under various transportation conditions. We identified the dominant mold strains responsible for soybean mildew. Using GC‐IMS technology, we analyzed the concentration changes of volatile substances during soybean mildew, pinpointing characteristic volatiles. Volatiles with large variations in concentration were identified as characteristic volatiles. This aids in accurately grading the soybean mildew levels.

## 2 MATERIALS AND METHODS

### 2.1 Materials

The soybean variety used in this experiment was Heinong No. 48, the predominant type in the Qiqihar area of Heilongjiang Province, China, during the harvest period. Heinong No. 48 is a high‐protein soybean variety and the main variety grown in the second cumulative temperature zone of Heilongjiang Province, which is produced by the Soybean Research Institute of the Heilongjiang Provincial Academy of Agricultural Sciences. Heinong No. 48 soybean has rounded grains with a yellow, glossy seed coat, a yellow umbilicus, and a hundred‐grain weight of about 22.0 g. It may cause moldy soybeans in storage and transport due to time and the environment, resulting in economic losses.

### 2.2 Simulated soybean container transportation conditions and identification of dominant molds

Soybean moisture content was adjusted to three groups: high (14 ± 0.5%), medium (13 ± 0.5%), and low (12 ± 0.5%). Each group, totaling 100 kg, was placed in containers (dimensions: 0.8 m length, 0.6 m width, 0.8 m height) and then placed in a constant temperature and humidity box (HS‐800, Shanghai Hesheng Instrument Technology Co., China) to replicate the varying temperature and humidity conditions of transportation environments. Simulated transportation temperatures were set to 20, 30, 40, 50 and 60°C, and relative humidity conditions were 30%, 50%, 70%, and 90%. Each temperature was treated at each humidity condition. Random sampling occurred at 0, 8, 16, 24, and 32 days, with each group sampled nine times (eight corners of containers and one center of the soybean pile). Concurrently, soybeans with different moisture contents were mixed with distilled water at a 1:5 ratio (water: soybeans, V/m) to simulate soybean condensation based on previous studies. Samples were taken at 0, 4, 8, 12, 16, 20, 24, 28, and 32 days to determine the total mold colonies in soybean samples. Mold colonies were quantified following GB 4789.15‐2016 National Food Safety Standard Food Microbiological Examination Mold and Yeast Count. The average value of all samples represented the final total number of mold colonies.

Grain safety were levels following LS/T6132‐2018 Inspection of grain and oils‐Storage fungal examination‐Enumeration spores of mold (LS/T6132‐2018). The total mold colonies in non‐moldy soybean was <1.0 × 10^5^ CFU/g. Mildly mildewed soybeans had 1.0–9.9 × 10^5^ CFU/g, moderately mildewed soybeans had 1.0–9.9 × 10^6^ CFU/g, and severely mildewed soybeans had ≥1.0 × 10^7^ CFU/g.

#### 2.2.1 Isolation of the main mold strains of moldy soybean

For analysis, 25 g of moldy soybean samples in a 500‐mL conical flask were mixed with 225 mL of distilled water at a 1:9 mass ratio. The mixture was shaken for 20 min on a constant temperature shaking table (TH2‐C; Taicang Experimental Equipment Factory, China) at 20°C and 150 rpm. Following this, 1 mL of supernatant was taken in a 25 ‐mL sterile test tube, mixed with 9 mL of distilled water, and uniformly diluted to a 10^−7^ gradient. Subsequently, 10^−5^, 10^−6^, and 10^−7^ gradient sample diluents were uniformly coated on Bengal red culture medium and placed in a mold incubator at 28 ± 1°C. The isolated single‐colony molds were sub‐cultured thrice, inoculated on the inclined plane of Bengal culture medium, and refrigerated at 4°C for later use.

#### 2.2.2 Morphological observation and ITS biological sequencing

The morphological changes of isolated single colony molds were observed, including characteristics like colony size, color, and mycelium growth throughout the growth process.

Internal Transcribed Spacer (ITS) identification refers to the DNA sequencing of ITS sequences, and by comparing the sequenced ITS sequences with known fungal ITS sequences, a method is used to obtain information about the fungal species to be tested. Referring to Yang, Fang, et al. (2021), Yang, Wang, et al. (2021) method, 20 mg of dried mold hyphae were pulverized with liquid nitrogen and added to a 1.5 mL sterile centrifuge tube. Genomic DNA extraction employed the SK8259‐Ezup column extraction kit, followed by PCR amplification (2720 Thermal Cycler; Applied Biosystems, USA). The reaction system details and conditions are outlined in Tables 1 and 2. Primer‐ITS1 and Primer‐ITS4 are fungal universals. The amplified products underwent 1% agarose electrophoresis (150 V, 100 mA, 20 min). The desired DNA bands were excised and sequenced using the SK8131‐SanPrep DNA gel recovery kit. Sequence comparison via BLAST on NCBI identified the sequences with the highest homology.

**TABLE 1:** PCR reactions.

Reaction ingredients	V (μL)
10× PCR buffer	/
dNTP (each 10 mM)	/
Taq Plus DNA polymerase (5 U/μL)	/
50 mM MgSO_4_	12.5
Primer‐ITS1 (TCCGTAGGTGAACCTGCGG)	1
Primer‐ITS4 (TCCTCCGCTTATTGATATGC)	1
Template (DNA)	1
ddH_2_O	9.5
Total	25

**TABLE 2:** PCR cycle conditions.

T (°C)	Time	Program
95	5 min	Pre‐denaturation
94	30 s	30 cycle
57	30 s	
72	90 s	
72	10 min	Repair extension
4	∞	Termination reaction

### 2.3 Identification of soybean mildew grade based on GC‐IMS technology

#### 2.3.1 Preparation of mold spore suspension

The isolated and screened dominant mold strains were cultured in Bengal red culture medium, placed in a constant temperature mold incubator (28 ± 1°C, 3–7 days) for activation, eluted with 0.9% sterile normal saline, counted using a blood cell counting plate (25 × 16), and adjusted to a spore suspension concentration of 1.0 × 10^6^–1.0 × 10^7^ CFU/mL for subsequent use.

#### 2.3.2 Preparation of moldy soybean samples

To eliminate external interference, it is essential to sterilize experimental soybeans using ultraviolet irradiation (Hidaka & Kubota, 2006). Spraying a spore suspension of dominant mold strains on 500 g samples of UV‐sterilized condensed soybeans at a 1:10 ratio (suspension: soybean, mL/g) is the prescribed method. Three soybean samples are inoculated with each dominant mold and cultured in a constant temperature and humidity box at 30°C with 90% relative humidity. Concurrently, to ensure test result accuracy, an additional 500 g of condensed soybeans without UV sterilization is placed in a culture box with a constant temperature and humidity for natural mildewing. Random sampling occurs at 0, 8, 16, and 24 days, with each group sampled thrice (10 g each time) and placed in a 20 ‐mL headspace sample bottle for subsequent use.

#### 2.3.3 GC‐IMS determination

Using GC‐IMS (FlavourSpec, Germany GAS) following the method described by Gu, Chen, et al. (2020), Gu, Wang, et al. (2020), the sample was hatched at 60°C for 15 min. A 1.0‐mL syringe (80°C) injected 500 μL of the sample into the port (85°C). The analyte underwent separation via the MXT‐5 chromatographic column under isothermal conditions. Subsequently, the analyte traversed the drift area through the shutter grille, finally entering the IMS detector. The tritium source (^3^H) served as the ionization source. The drift tube, measuring 5 cm, maintained a drift field of 400 V/cm. The IMS operated in positive reactant ion mode. The volatile compound retention index (RI) was calculated using N‐ketone mixtures as an external reference (Speckbacher et al., 2021). Specific detection conditions are detailed in Tables 3 and 4. Volatile substances were qualitatively analyzed by searching the NIST and IMS databases.

**TABLE 3:** GC condition.

Time	EPC1 (Drift gas flow)	EPC2 (Carrier gas flow)	Record
00:00,000	150 mL/min	2 mL/min	‐Rec
02:00,000	150 mL/min	2 mL/min	—
20:00,000	150 mL/min	100 mL/min	‐Stop

**TABLE 4:** Instrument parameter condition.

	Condition	Parametric
Auto‐headspace inlet sampler	Injection volume	500 μL
	Incubation time	15 min
	Incubation temperature	80°C
	Temperature of the injection needle	85°C
	Incubation speed	500 rpm
GC‐IMS	Analysis time	20 min
	Column type	MXT‐5, 15 mL, 0.53 mm ID, 1 μm FT
	Column temperature	60°C
	Gas/drift gas	N_2_ (99.999%)
	IMS temperature	45°C

### 2.4 Data analysis

The VOCal software (GAS Co., Dortmund, Germany), utilizing the NIST and IMS databases, facilitates qualitative analysis of detected VOCs. For visual analysis of sample determination data, the Reporter, Gallery Plot, and Dynamic Principal Component Analysis (PCA) plug‐ins are employed. Data analysis and processing are conducted using IBM SPSS Statistics 26 software (International Business Machines Corporation).

## 3 RESULTS AND ANALYSIS

### 3.1 Simulation of the mildew law of soybeans under different transportation conditions

#### 3.1.1 Mildew regularity of uncondensed soybean

During transportation, the container's temperature and humidity primarily hinge on soybean respiration and microbial metabolism on the soybean grain surface. With low soybean moisture content, both soybean grain and surface microorganism respiration weaken, maintaining a balanced state of temperature and humidity. Conversely, higher soybean water content intensifies grain respiration. The greater the water content, the stronger the respiration, leading to increased container temperature and humidity, microbial proliferation, and soybean mold.

**FIGURE 1:**
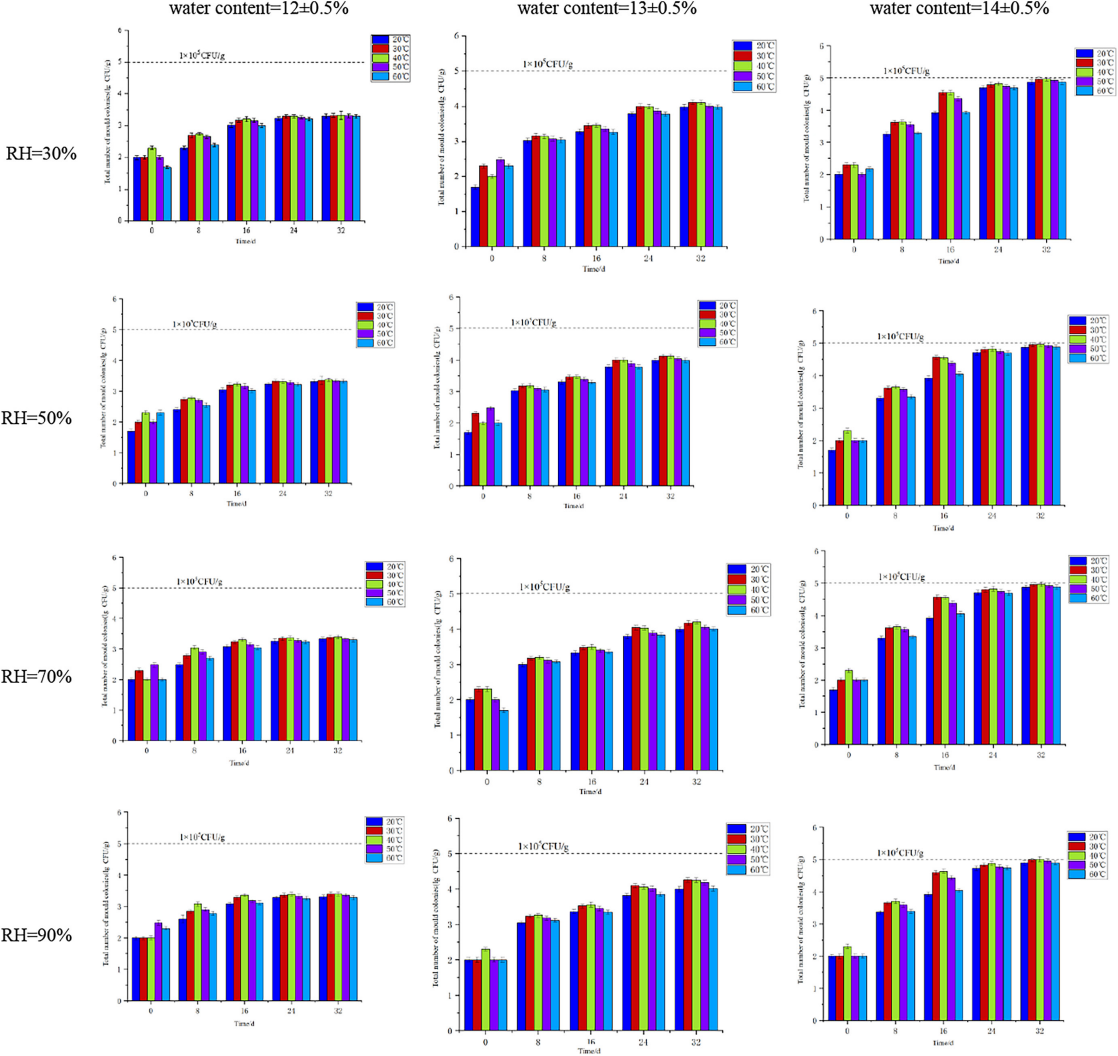
Changes in the total number of molds in soybeans with different moisture levels under different temperature and humidity conditions.

Figure 1 illustrates that, without condensation, the total mold count trend in high‐moisture soybeans resembles that in low‐moisture and medium‐moisture soybeans. However, the total mold count rises noticeably faster than in low‐moisture and medium‐moisture soybeans. Between 24 and 32 days, the total mold count in high‐moisture soybeans nears the safety limit. By day 32, at 40°C and RH = 90%, the total mold count in soybean samples reached 1.0 × 10^5^ CFU/g, signifying potential soybean mildew under these temperature and humidity conditions during transportation.

#### 3.1.2 Changes in total soybean mold count in different moisture contents of condensed soybean

Soybeans, rich in fat and protein, exhibit substantial moisture absorption and desorption, influenced by environmental conditions. The transportation of grain from the north to the south faces temperature variations, creating a significant temperature contrast inside and outside the container. This temperature difference prompts micro‐airflow, gradually saturating the water vapor between bean grains within the container. As the warmer air meets the cooler bean pile, the saturated water vapor between the grains cools and condenses into liquid water on the bean surface. The likelihood of condensation increases with a larger ambient temperature difference. This condensation results in a rapid localized moisture surge in soybean heaps, creating an environment conducive to mold growth. Failure to implement timely measures can culminate in soybean mold.

**FIGURE 2:**
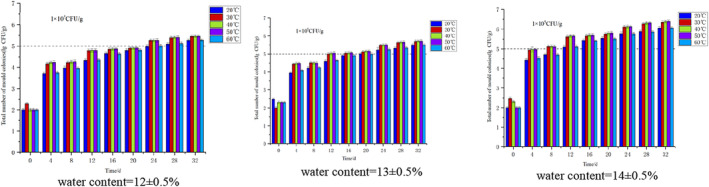
Changes in the total number of molds in soybeans under different temperature and humidity conditions under dewing.

Figure 2 illustrates that, during condensation, the total mold count significantly rises with prolonged transportation time for high, medium, and low‐moisture soybeans. Higher moisture levels correlate with a faster mold growth rate. Notably, soybeans with high, medium, and low moisture content exhibit mold presence of 1.0 × 10^5^ CFU/g at 4, 12, and 24 days, respectively, signaling the onset of mildew. High‐moisture soybeans manifest severe mildew at 24 days, with a total mold count reaching 1.0 × 10^6^ CFU/g. This underscores that soybeans are prone to mildew under condensation conditions, with increased water content accelerating the process.

### 3.2 Screening and identification of dominant mold strains of moldy soybean

#### 3.2.1 Screening and biological identification results of dominant mold strains

**FIGURE 3:**
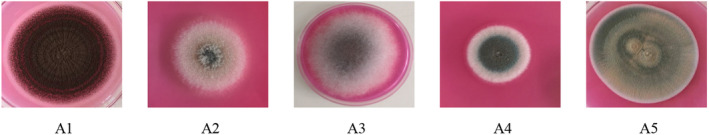
Observation of mold shape in mildewed soybean.

In the investigation of soybean mildew under varied transportation conditions, microorganisms isolated from differently affected soybeans (Figure 3) revealed common traits among the five main molds, including rapid growth and heightened spore yield. These microorganisms are preliminarily identified as the primary agents responsible for soybean mildew.

The ITS sequences of five molds were compared using BLAST on NCBI. Sequences showing the highest homology were selected as references. A1–A5 exhibited the highest homology with *Aspergillus niger* (MH511143.1), *Penicillium rubens* (MT765110.1), *Rhizopus microsporus* (MT672584.1), *Penicillium oxalicum* (MT795727.1), and *Aspergillus versicolor* (MN416222.1), respectively, with a 100% similarity. Based on these results and relevant literature (Liu, Bai, & Chen, 2017; Liu, Deng, et al., 2017), the primary mold strains in moldy soybeans were identified as *Aspergillus niger*, *Penicillium rubens*, *Rhizopus microsporus*, *Penicillium oxalicum*, and *Aspergillus versicolor*.

#### 3.2.2 Identification of soybean mildew grade based on GC‐IMS3.3.1 analysis of mVOCs of five dominant molds

Different molds produce distinct mVOCs during growth and metabolism. Complex mVOCs were continuously detected with varying mobility. Therefore, according to the ion migration time and ion peak intensity differences among the component ions, the corresponding mVOCs of five dominant mold strains were analyzed.

##### Qualitative analysis of the mVOCs of five dominant molds

Sample analyte separation relies on retention time/retention index (*y*‐axis) and drift time (RIP)/ion mobility (*x*‐axis). RIP serves as a signal for water in air ionized by tritium‐beta radiation, commonly used as a reference signal indicating the total number of ions available for ionization. By discerning ion signal strength differences, volatile substances with clear distinctions can be highlighted (Chen et al., 2020). Additionally, the same volatile substances may generate various signals, such as protonated monomers (M) and proton‐bound dimers (D). This characteristic can be attributed to substances with high proton affinity or signals enabling ions to form dimers during drift cell movement (Thomas et al., 2021).

**FIGURE 4:**
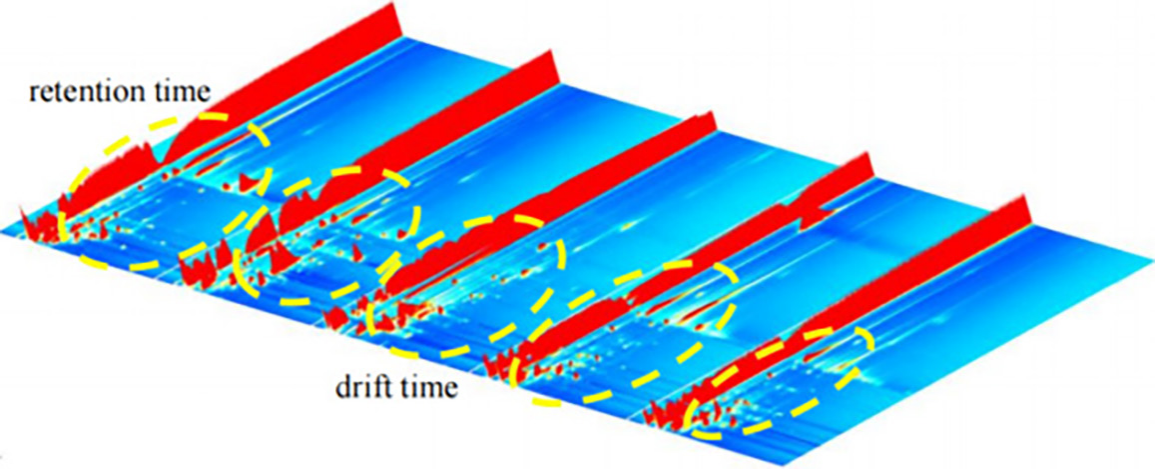
Three‐dimensional topographic map of five dominant mold mVOCs.

Figure 4, presents a three‐dimensional mVOC spectrum detected via GC‐IMS. The *x*‐axis denotes ion migration time, the *y*‐axis represents gas chromatograph retention time, and the *z*‐axis represents peak intensity. From the three‐dimensional map, we can intuitively see the differences in mVOCs in different mold samples. Examining the figure, differences in peak signal intensities among the five predominant molds become apparent in mVOCs, indicating variations in mVOC concentrations.

To analyze the distinctions in mVOCs among five prevalent mold strains, we employed a two‐dimensional mobility spectrogram. The spectrum background is blue, with a red vertical line at Abscissa 1.0 indicating the RIP peak. By assessing the presence or absence of peaks (color points) or color depth, we can visually convey composition and concentration differences between samples. Each point flanking the RIP peak signifies a volatile organic compound, with color denoting substance concentration—white indicates lower concentration, red indicates higher concentration, and darker colors indicate even higher concentration (Gallegos et al., 2017). To further compare the differences in mVOCs of five dominant mold strains, the mVOCs of *Aspergillus niger* were used as reference samples. The background is white, with blue indicating concentrations below the reference, while red signifies concentrations surpassing the reference.

**FIGURE 5:**
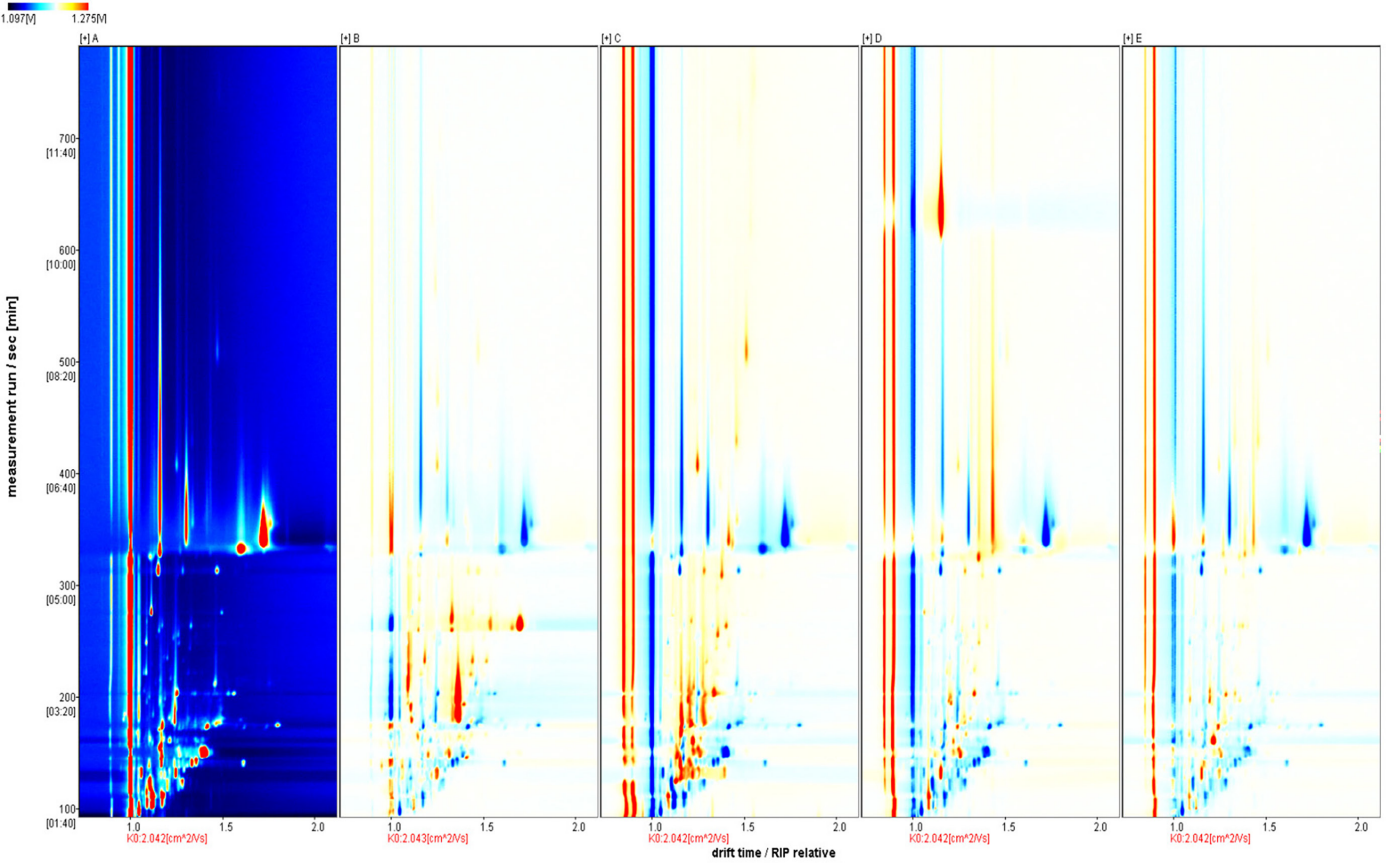
Differential spectra of mVOCs of five fungal strains.

In Figure 5, the fingerprint area displays mVOC retention times for the five dominant mold strains, ranging from 100 to 500 s, with drift times between 1.0 and 1.8 ms. Most differential mVOCs cluster between 100 and 400 s, suggesting that low‐molecular‐weight volatile substances (RI < 400) primarily contribute to mold‐specific mVOCs, serving as distinctive markers for mold identification.

##### Characteristic volatile fingerprint analysis

**FIGURE 6:**
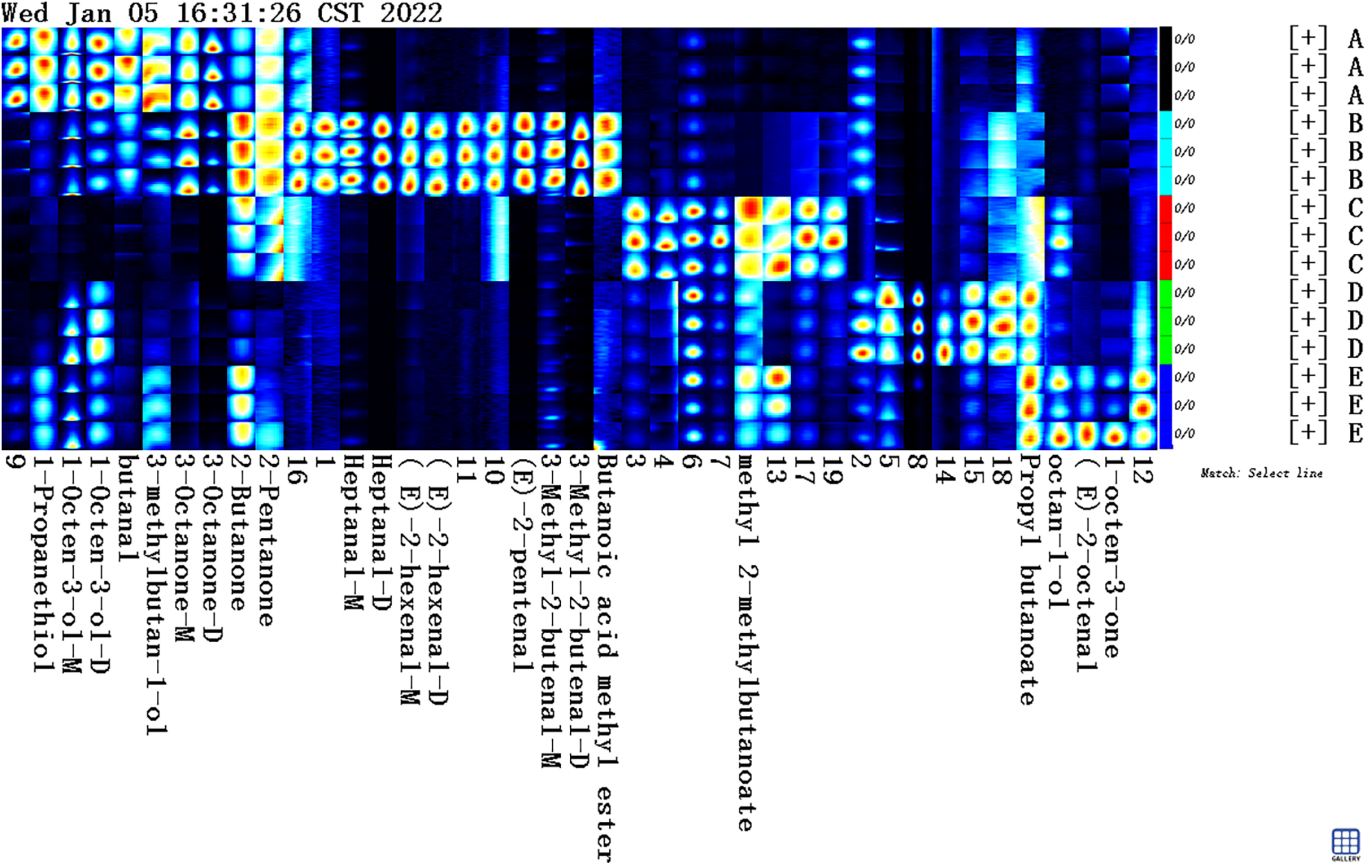
Gallery plot showing fingerprints of five dominant fungal strains.

Three‐dimensional mobility spectrograms and two‐dimensional difference spectrograms can only illustrate overall differences in mVOCs among different molds. They cannot precisely determine specific VOCs. Thus, a detailed fingerprint analysis utilizes all signal peaks. In the fingerprint, each row corresponds to a sample's signal peak, while each column represents a substance. Brightness indicates signal strength, with a brighter signal indicating stronger strength and a darker signal indicating weaker strength (Han et al., 2022). Figure 6 directly reveals obvious differences among the mVOCs of the five dominant mold strains. Each mold exhibits characteristic mVOCs, facilitating mold identification (Table 5 has related mVOC information). Propyl butyrate and 2‐butanone are consistently produced during the growth and metabolism of the five dominant mold strains. *Aspergillus niger*, *Penicillium rubricogenes*, *Penicillium oxalate*, and *Aspergillus versicolor* all produce 1‐octene‐3‐ol. *Rhizopus microsporus*, *Penicillium oxalicum*, and *Aspergillus versicolor* all generate methyl 2‐methylbutyrate. *Aspergillus niger*, *Penicillium rubrinogenes*, and *Aspergillus versicolor* produce 3‐methylbutan‐1‐ol. Both *Aspergillus niger* and *Penicillium rubrinogenes* can produce 3‐octanone and butyraldehyde. *Aspergillus niger* and *Aspergillus versicolor* share the production of 1‐propanethiol. *Aspergillus versicolor* is distinctive in producing 1‐octene‐3‐one and (E)‐2‐octenal. *Penicillium rubra* produces unique mVOCs, including methyl butyrate, 3‐methyl‐2‐butenal, (E)‐2‐pentenal, (E)‐2‐hexenal, and heptanal. These results demonstrate differing characteristic mVOCs during the growth and metabolism of the five dominant molds. This distinction serves as a foundation for further exploration of specific VOCs for soybean mildew identification.

**TABLE 5:** Characteristic mVOCs information table of 5 dominant molds.

Count	Compound	CAS#	Formula	MW	RI	Rt [s]	Dt [a.u.]
1	(E)‐2‐pentenal‐D	C1576870	C5H8O	84.1	746.8	183.888	1.36224
2	(E)‐2‐octenal	C2548870	C8H14O	126.2	1055.9	431.277	1.3353
3	Heptanal‐D	C111717	C7H14O	114.2	903.3	266.429	1.7042
4	Heptanal‐M	C111717	C7H14O	114.2	900.8	264.257	1.33162
5	3‐Methyl‐2‐butenal‐M	C107868	C5H8O	84.1	779.9	197.305	1.09076
6	3‐Methyl‐2‐butenal‐D	C107868	C5H8O	84.1	792.9	203.702	1.3632
7	Butanal	C123728	C4H8O	72.1	552.5	123.578	1.28301
8	(E)‐2‐hexenal‐M	C6728263	C6H10O	98.1	849.1	233.849	1.18394
9	(E)‐2‐hexenal‐D	C6728263	C6H10O	98.1	848.2	233.359	1.52042
10	3‐Octanone‐D	C106683	C8H16O	128.2	991.4	341.545	1.72187
11	3‐Octanone‐M	C106683	C8H16O	128.2	997.8	347.778	1.30584
12	1‐octen‐3‐one	C4312996	C8H14O	126.2	979.3	331.261	1.68922
13	2‐Butanone	C78933	C4H8O	72.1	580.2	131.061	1.24729
14	2‐Pentanone	C107879	C5H10O	86.1	685	159.326	1.12125
15	3‐methylbutan‐1‐ol	C123513	C5H12O	88.1	731.5	177.703	1.47766
16	1‐Octen‐3‐ol‐M	C3391864	C8H16O	128.2	991.8	341.935	1.16194
17	1‐Octen‐3‐ol‐D	C3391864	C8H16O	128.2	984.5	335.703	1.603
18	Octan‐1‐ol	C111875	C8H18O	130.2	1054.9	429.965	1.46068
19	1‐Propanethiol	C107039	C3H8S	76.2	625.3	143.208	1.35857
20	Propyl butanoate	C105668	C7H14O2	130.2	897.7	261.638	1.26816
21	Methyl 2‐methylbutanoate	C868575	C6H12O2	116.2	780.1	197.382	1.19617
22	Butanoic acid methyl ester	C623427	C5H10O2	102.1	703.9	166.48	1.4383

##### PCA of five dominant mold mVOCs

**FIGURE 7:**
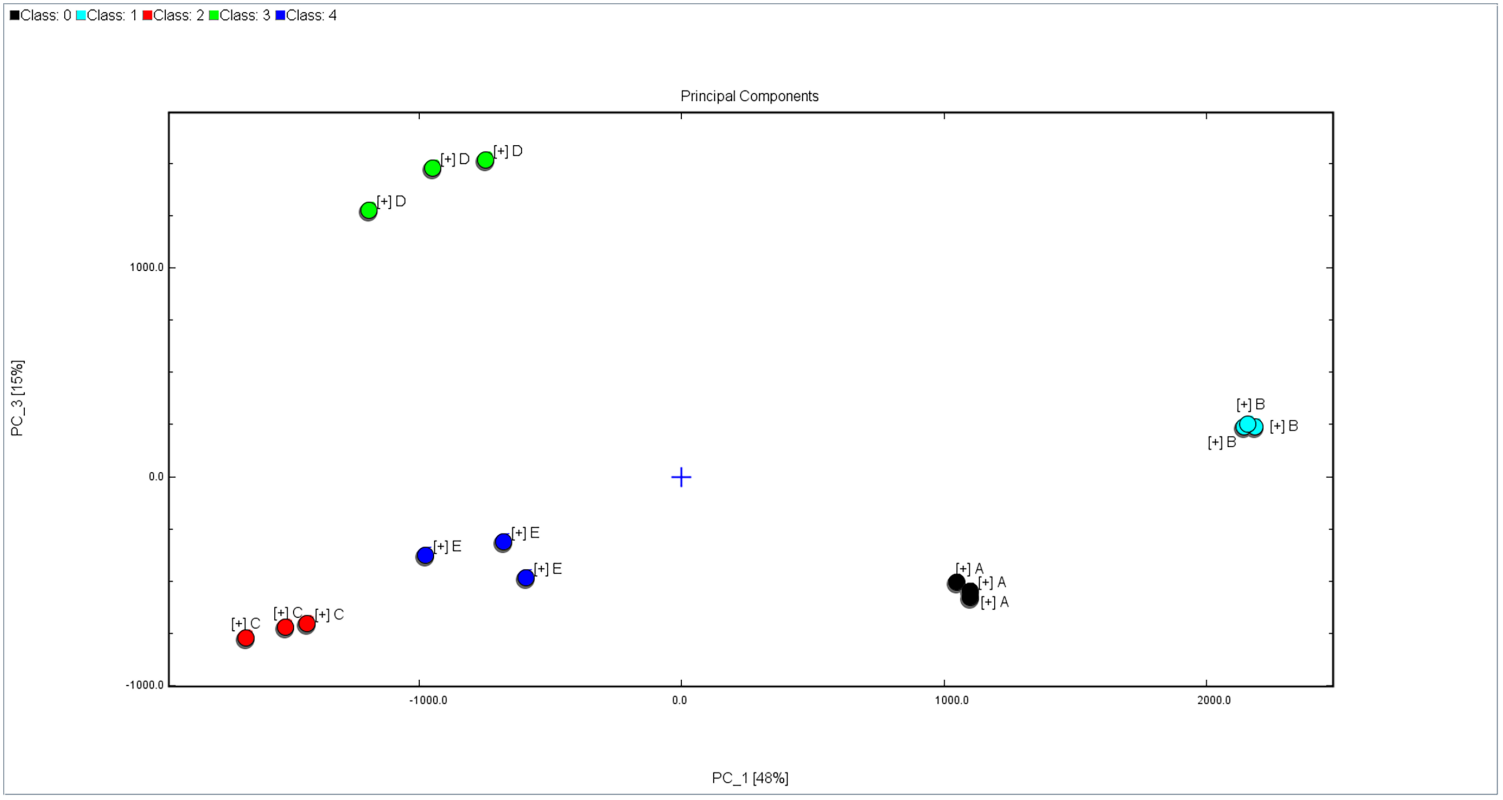
PCA of five dominant mold mVOCs.

PCA, a multivariate statistical detection method, utilizes the peak signal intensity of VOCs to highlight the differences between different molds. In Figure 7, the PCA outcomes for the 5 primary mold mVOCs are presented. Notably, the cumulative contribution rate of the first and third principal components amounts to 63.51%. These results signify the independence of the sample space in the PCA distribution map, enabling clear differentiation of the five dominant molds. Their characteristic mVOCs exhibit significant dissimilarities.

#### 3.2.3 Analysis of volatile substances in moldy soybeans

##### Qualitative analysis of volatile substances in moldy soybean

**FIGURE 8:**
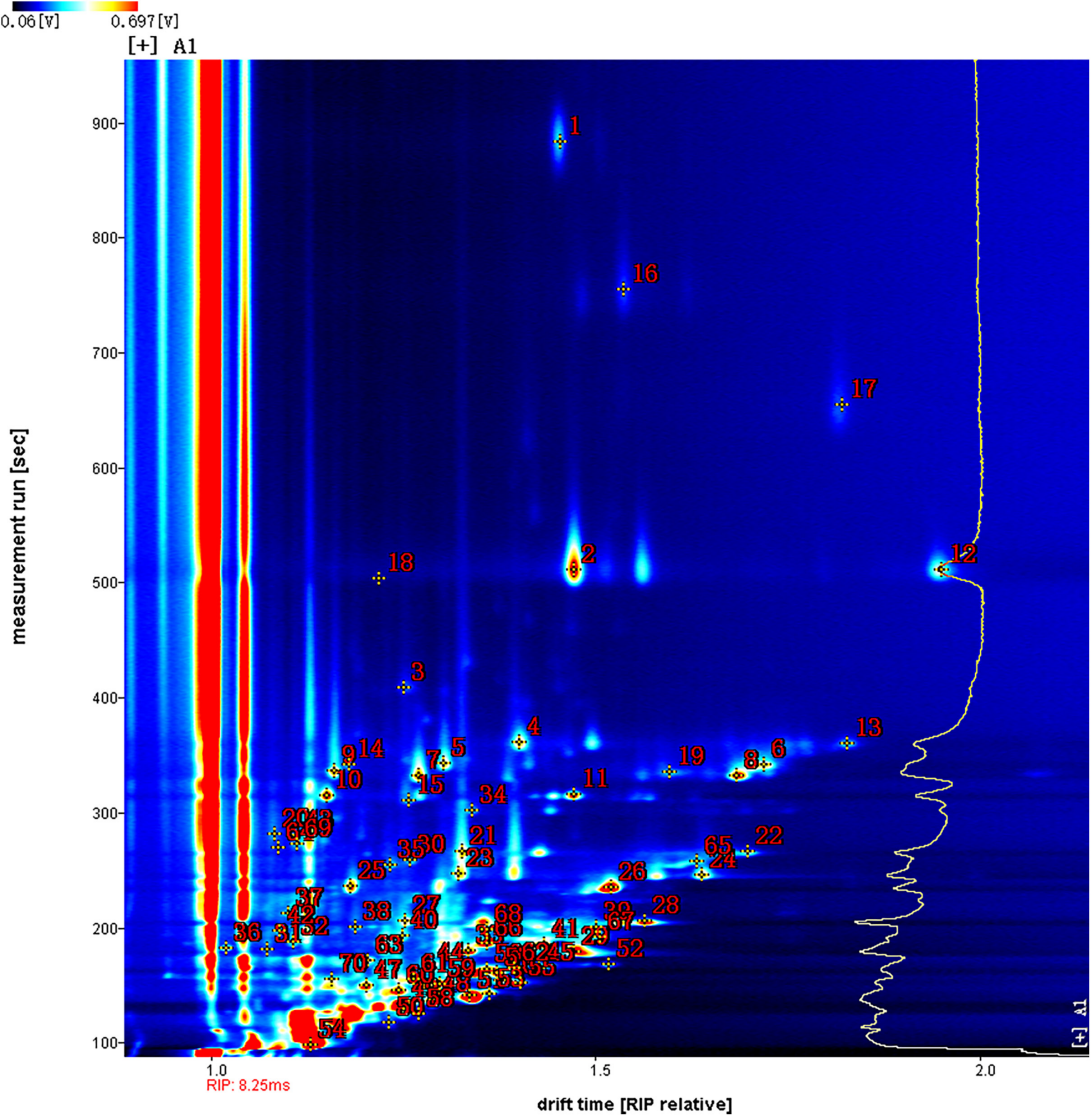
Ion migration spectra of mildewed soybean samples.

Compared to the mVOCs generated by an individual mold during the metabolic process, volatile substances from moldy soybean samples exhibit more complex compositions. To analyze and detect the VOCs in the soybean mildew process, the GC‐IMS library search plug‐in software characterized relevant compounds by comparing them with reference values of retention time (Rt) and drift time (Dt). In Figure 8, the horizontal and vertical axes are used to represent Dt and Rt, respectively, with each numerically marked point signifying an identified volatile organic compound. Figure 8 reveals that VOC signals in moldy soybeans predominantly manifest in the retention time range of 100–800 s and the drift time range of 1.0–1.8 ms, detecting a total of 54 compounds. Specific information, including 24 aldehydes, 15 ketones, 10 alcohols, 2 esters, 1 pyrazine, and 1 aromatic compound, can be found in Table 6. Notably, aldehydes, ketones, and alcohols constitute a significant portion of these compounds. Additionally, it is observed that some individual compounds generate multiple signal points, primarily formed by dimers with relatively high concentrations in IMS drift tubes.

**TABLE 6:** Information table of volatile substances in mildewed soybean.

Type	Count	Compound	CAS#	Formula	MW	RI	Rt [s]	Dt [a.u.]
Aldehydes	2	Nonanal‐M	C124196	C_9_H_18_O	142.2	1111.5	511.348	1.47389
	3	Benzene acetaldehyde	C122781	C_8_H_8_O	120.2	1040.3	408.9	1.25179
	4	Octanal‐M	C124130	C_8_H_16_O	128.2	1007.2	361.285	1.40241
	10	Benzaldehyde‐M	C100527	C_7_H_6_O	106.1	960.2	314.923	1.15239
	11	Benzaldehyde‐D	C100527	C_7_H_6_O	106.1	960.9	315.518	1.47302
	12	Nonanal‐D	C124196	C_9_H_18_O	142.2	1111.6	511.436	1.95149
	13	Octanal‐D	C124130	C_8_H_16_O	128.2	1006.5	360.349	1.8289
	15	(E)‐hept‐2‐enal	C18829555	C_7_H_12_O	112.2	954.8	310.389	1.25794
	16	Decanal	C112312	C_10_H_20_O	156.3	1281.4	755.616	1.53806
	21	Heptanal‐M	C111717	C_7_H_14_O	114.2	902.7	265.848	1.32731
	22	Heptanal‐D	C111717	C_7_H_14_O	114.2	903	266.137	1.69917
	25	(E)‐2‐hexenal‐M	C6728263	C_6_H_10_O	98.1	852.6	235.73	1.18333
	26	(E)‐2‐hexenal‐D	C6728263	C_6_H_10_O	98.1	851.6	235.151	1.52163
	27	Hexanal‐M	C66251	C_6_H_12_O	100.2	796.4	205.613	1.25462
	28	Hexanal‐D	C66251	C_6_H_12_O	100.2	797	205.903	1.56496
	32	(E)‐2‐pentenal‐M	C1576870	C_5_H_8_O	84.1	755	187.211	1.10771
	42	3‐methyl‐2‐butenal‐M	C107868	C_5_H_8_O	84.1	779.7	197.247	1.09103
	43	(E,E)‐2,4‐hexadienal	C142836	C_6_H_8_O	96.1	919.8	280.48	1.11539
	45	Pentanal	C110623	C_5_H_10_O	86.1	699.8	164.832	1.42865
	55	3‐Methylbutanal	C590863	C_5_H_10_O	86.1	658.2	152.1	1.404
	64	3‐Methylthiopropanal	C3268493	C_4_H_8_OS	104.2	907.1	269.665	1.08918
	66	(E)‐2‐pentenal‐D	C1576870	C_5_H_8_O	84.1	752.5	186.203	1.36065
	68	3‐Methyl‐2‐butenal‐D	C107868	C_5_H_8_O	84.1	782.6	198.421	1.3598
	70	2‐Methylbutanal	C96173	C_5_H_10_O	86.1	671.8	155.772	1.15929
Ketone	5	3‐Octanone‐M	C106683	C_8_H_16_O	128.2	993.1	343.064	1.30469
	6	3‐Octanone‐D	C106683	C_8_H_16_O	128.2	991.9	341.978	1.72127
	7	1‐Octen‐3‐one‐M	C4312996	C_8_H_14_O	126.2	979.9	331.75	1.27103
	8	1‐Octen‐3‐one‐D	C4312996	C_8_H_14_O	126.2	980.9	332.644	1.6857
	14	Methyl‐5‐hepten‐2‐one	C110930	C_8_H_14_O	126.2	992	342.103	1.18039
	20	Dihydro‐2(3h)‐furanone	C96480	C_4_H_6_O_2_	86.1	920.9	281.447	1.08493
	30	2‐Heptanone‐M	C110430	C_7_H_14_O	114.2	894.4	258.818	1.26103
	37	Cyclopentanone	C120923	C_5_H_8_O	84.1	808.9	212.306	1.10272
	38	2‐Kexanone‐M	C591786	C_6_H_12_O	100.2	786.5	200.307	1.18992
	39	2‐Kexanone‐D	C591786	C_6_H_12_O	100.2	786.5	200.307	1.50229
	49	2‐Butanone	C78933	C_4_H_8_O	72.1	591.1	133.996	1.25225
	56	3‐Pentanone	C96220	C_5_H_10_O	86.1	696.8	163.62	1.35998
	57	2‐Pentanone	C107879	C_5_H_10_O	86.1	689	160.467	1.37319
	65	2‐Heptanone‐D	C110430	C_7_H_14_O	114.2	893.1	257.689	1.63354
	69	Cyclohexen‐2‐one	C930687	C_6_H_8_O	96.1	910.6	272.598	1.11297
Alcohols	9	1‐Octen‐3‐ol‐M	C3391864	C_8_H_16_O	128.2	984.9	336.069	1.16201
	19	1‐Octen‐3‐ol‐D	C3391864	C_8_H_16_O	128.2	983.8	335.096	1.59817
	23	n‐Hexanol‐M	C111273	C_6_H_14_O	102.2	874.2	247.314	1.32312
	24	n‐Hexanol‐D	C111273	C_6_H_14_O	102.2	872.1	246.155	1.64045
	29	2‐Methylbutan‐1‐ol	C137326	C_5_H_12_O	88.1	735.4	179.26	1.4741
	40	Pentan‐1‐ol‐M	C71410	C_5_H_12_O	88.1	769.7	193.164	1.2498
	46	1‐Butanol	C71363	C_4_H_10_O	74.1	664.4	153.763	1.37799
	53	2‐Methyl‐1‐propanol	C78831	C_4_H_10_O	74.1	623.3	142.693	1.36442
	54	Ethanol	C64175	C_2_H_6_O	46.1	457.2	97.89	1.13097
	67	Pentan‐1‐ol‐D	C71410	C_5_H_12_O	88.1	767.7	192.381	1.50743
Esters	1	2‐Butenoic acid, hexyl ester	C19089920	C_10_H_18_O_2_	170.3	1370.1	883.109	1.45612
	34	Propyl isovalerate	C557006	C_8_H_16_O_2_	144.2	945	301.99	1.34134
	51	Ethyl acetate	C141786	C_4_H_8_O_2_	88.1	616.3	140.796	1.33819
Pyrazine	18	2‐Ethyl‐3,5‐dimethylpyrazine	C27043056	C_8_H_12_N_2_	136.2	1106.1	503.555	1.22034
Aromatic compounds	36	Toluene	C108883	C_7_H_8_	92.1	743.5	182.549	1.02094

##### Fingerprinting analysis of volatile substances in moldy soybeans

**FIGURE 9:**
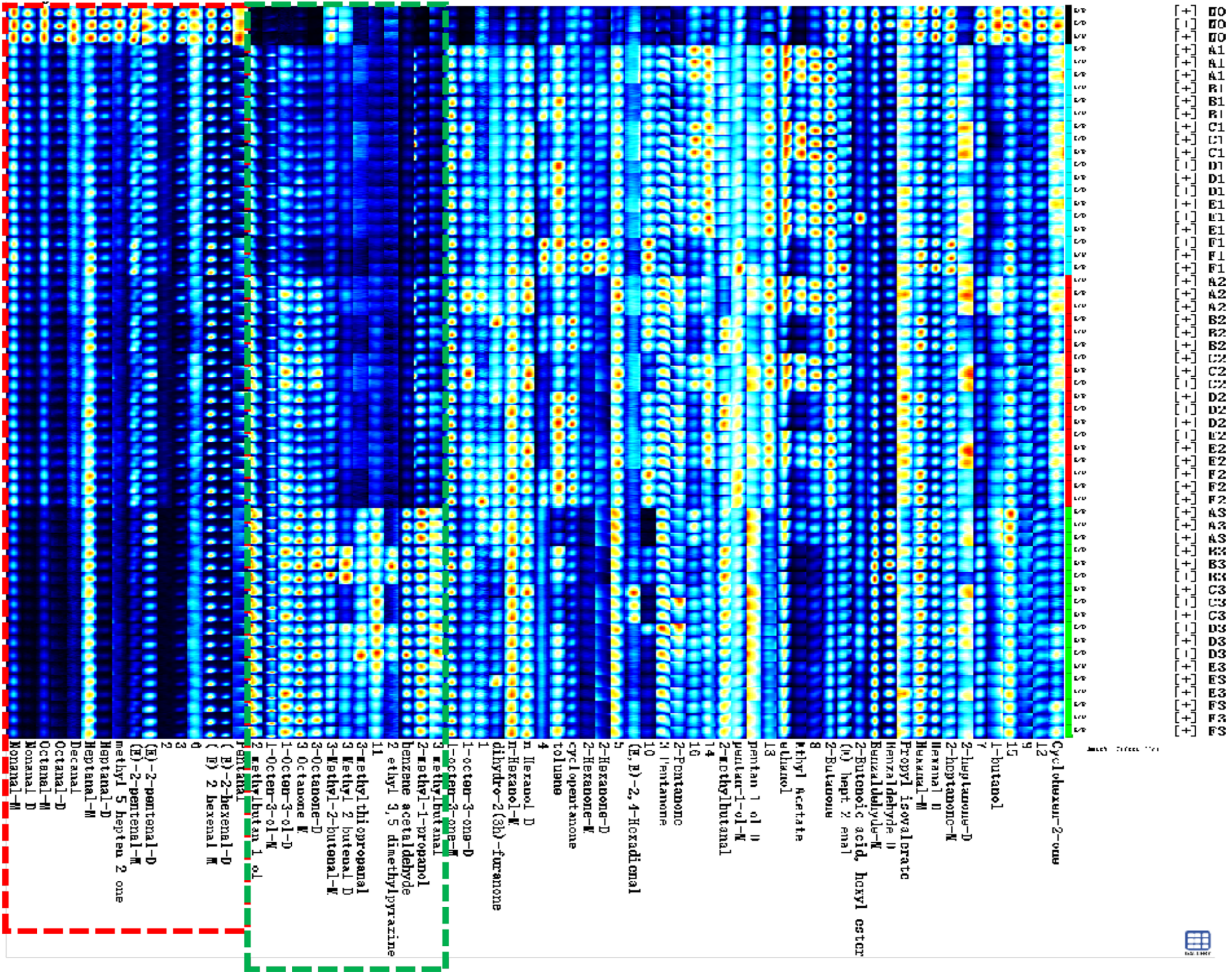
Gallery plot fingerprints of moldy soybean samples.

To intuitively compare representative signal peaks of soybean samples with varying mildew degrees, the Gallery Plot plug‐in facilitated the visualization of fingerprint spectra (Figure 9). Notably, as mildew intensified, the concentrations of nonanal, n‐octanal, decanal, heptanal, valeraldehyde, 6‐methyl‐5‐heptene‐2‐one, (E)‐2‐hexenal, and (E)‐2‐pentenal steadily decreased. These compounds are unique volatile substances in healthy soybeans. The deepening of mildew led to the microbial metabolism of soybean nutrients (amino acids, fatty acids), resulting in a reduction of these original volatile substances. Simultaneously, phenylacetaldehyde, 3‐methyl‐2‐butenal, 3‐methylbutyraldehyde, 3‐methylthio‐propanal, 3‐octanone, 1‐octen‐3‐ol, 2‐methyl‐1‐propanol, 2‐methylbutan‐1‐ol, and 2‐ethyl‐3,5‐dimethylpyrazine exhibited a gradual increase with the progression of mildew. This pattern suggests that certain VOCs are characteristic markers produced by dominant molds during their growth and metabolism. Examples include 3‐octanone, 1‐octene‐3‐ol, and 3‐methyl‐2‐butenal (as determined from the 3.3.1 analysis). Moreover, specific VOCs, such as 3‐methylthiopropanal, 3‐methylbutyraldehyde, 2‐ethyl‐3,5‐dimethylpyrazine, are indicative of dominant molds utilizing soybean as a growth substrate.

As shown in Table 7, the results indicate significant variations in the composition of VOCs among soybean samples with varying degrees of mildew. However, the composition of VOCs in soybean samples inoculated with different molds remains similar at the same mildew degree. This similarity could arise from the shared metabolites of the five dominant molds utilizing soybean as a growth substrate or the limited total mold count, preventing the accumulation of distinct VOCs to an observable extent.

**TABLE 7:** Compound information table of 18 characteristic points of moldy soybean.

Count	Compound	CAS#	Formula	MW	RI	Rt [s]	Dt [a.u.]
1	Nonanal‐M	C124196	C_9_H_18_O	142.2	1111.5	511.348	1.47389
2	Octanal‐M	C124130	C_8_H_16_O	128.2	1007.2	361.285	1.40241
3	Nonanal‐D	C124196	C_9_H_18_O	142.2	1111.6	511.436	1.95149
4	Octanal‐D	C124130	C_8_H_16_O	128.2	1006.5	360.349	1.8289
5	6‐methyl‐5‐hepten‐2‐one	C110930	C_8_H_14_O	126.2	992	342.103	1.18039
6	Decanal	C112312	C_10_H_20_O	156.3	1281.4	755.616	1.53806
7	Heptanal‐M	C111717	C_7_H_14_O	114.2	902.7	265.848	1.32731
8	Heptanal‐D	C111717	C_7_H_14_O	114.2	903	266.137	1.69917
9	(E)‐2‐hexenal‐M	C6728263	C_6_H_10_O	98.1	852.6	235.73	1.18333
10	(E)‐2‐hexenal‐D	C6728263	C_6_H_10_O	98.1	851.6	235.151	1.52163
11	(E)‐2‐pentenal‐M	C1576870	C_5_H_8_O	84.1	755	187.211	1.10771
12	Pentanal	C110623	C_5_H_10_O	86.1	699.8	164.832	1.42865
13	(E)‐2‐pentenal‐D	C1576870	C_5_H_8_O	84.1	752.5	186.203	1.36065
14	Benzene acetaldehyde	C122781	C_8_H_8_O	120.2	1040.3	408.9	1.25179
15	3‐Octanone‐M	C106683	C_8_H_16_O	128.2	993.1	343.064	1.30469
16	3‐Octanone‐D	C106683	C_8_H_16_O	128.2	991.9	341.978	1.72127
17	1‐Octen‐3‐ol‐M	C3391864	C_8_H_16_O	128.2	984.9	336.069	1.16201
18	2‐Ethyl‐3,5‐dimethylpyrazine	C27043056	C_8_H_12_N_2_	136.2	1106.1	503.555	1.22034
19	1‐Octen‐3‐ol‐D	C3391864	C_8_H_16_O	128.2	983.8	335.096	1.59817
20	2‐Methylbutan‐1‐ol	C137326	C_5_H_12_O	88.1	735.4	179.26	1.4741
21	3‐Methyl‐2‐butenal‐M	C107868	C_5_H_8_O	84.1	779.7	197.247	1.09103
22	2‐Methyl‐1‐propanol	C78831	C_4_H_10_O	74.1	623.3	142.693	1.36442
23	3‐Methylbutanal	C590863	C_5_H_10_O	86.1	658.2	152.1	1.404
24	3‐Methylthiopropanal	C3268493	C_4_H_8_OS	104.2	907.1	269.665	1.08918
25	3‐Methyl‐2‐butenal‐D	C107868	C_5_H_8_O	84.1	782.6	198.421	1.3598

*Note*: Red and green marks indicate VOCs with positive and negative correlation trends with the mildew degree, respectively.

##### PCA and cluster analysis of VOCs in soybeans with different mildew degrees

**FIGURE 10:**
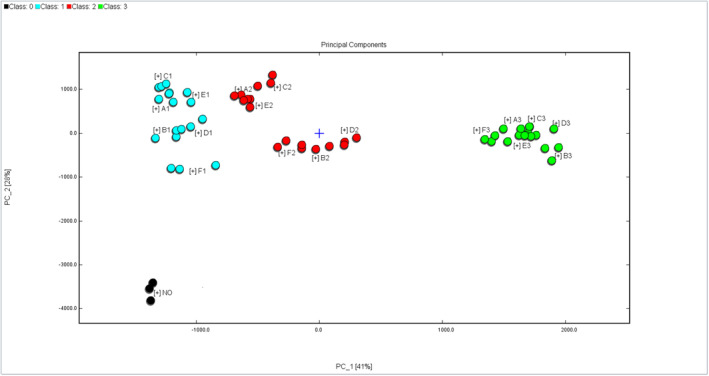
PCA score map of mildewed soybean samples.

For PCA analysis, we selected the peak signal intensity of VOCs in moldy soybean samples. As shown in the left side of Figure 10, the cumulative contribution rate of the first and second principal components reaches 69.81%. The distribution of soybean samples with different mildew degrees appears relatively independent, demonstrating PCA's effectiveness in distinguishing soybean samples with varying mildew levels. Notably, the differentiation between mildly and severely mildewed soybean samples is minimal, with considerable overlap among soybean samples contaminated by different molds at the same mildew degree. This overlap may be attributed to the small total mold count in mildly mildewed soybean samples, preventing the accumulation of distinct VOCs to obviously expressed degrees. Additionally, there could be partial overlap in the VOCs produced by different molds using soybean as a growth substrate, influencing the differentiation effect.

**FIGURE 11:**
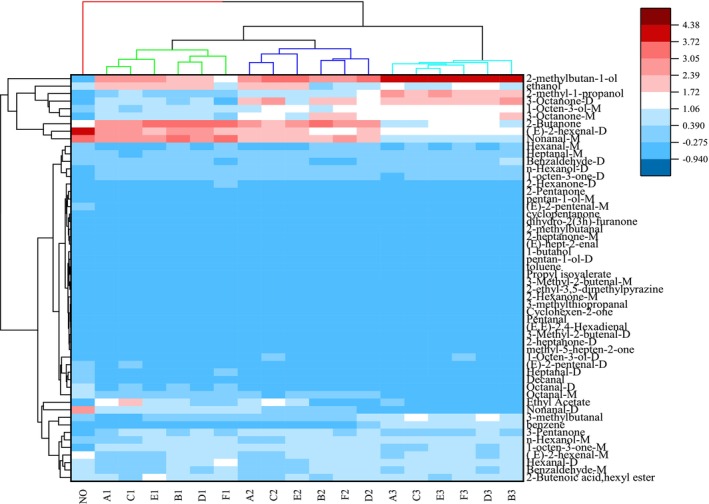
Cluster analysis of volatile organic compounds in moldy soybean.

To examine variations in VOCs among soybean samples with varying degrees of mildew, we conducted cluster analysis. In Figure 10, a strong correlation is observed between soybean samples with different mildew levels along the horizontal axis and VOCs along the vertical axis within the same category. As the Euclidean distance decreases, the correlation between samples increases (Yang, Fang, et al., 2021; Yang, Wang, et al., 2021). Cluster analysis categorized moldy soybean samples into four groups: no mildew, mild mildew, moderate mildew, and severe mildew. Soybean samples with the same mildew degree exhibited a substantial correlation. Figure 11 illustrates that unmolded soybeans display elevated levels of certain VOCs, like nonanal, octanal, and decanal. Conversely, as mildew deepens, the levels of these compounds gradually decline, while other VOCs, including 3‐methylbutyraldehyde, 3‐methylthiopropionaldehyde, 3‐octanone, and 1‐octen‐3‐ol, progressively increase. Employing a horizontal model, moldy soybean VOCs can be categorized into two groups based on their change trend (increase and decrease), with the corresponding compounds displaying consistent trends. The outcomes reveal significant disparities in VOCs among soybean samples with varying mildew degrees, and the VOC content fluctuates with the degree of mildew. The results were similar to those from fingerprint spectrum and PCA analysis, providing further validation for the viability of identifying VOCs in soybean samples with different mildew degrees through GC‐IMS.

## 4 CONCLUSION

To safeguard the quality and safety of soybean bulk grain during extended container transport, we examined the primary molds responsible for soybean mildew by simulating under varying transportation conditions and analyzing the change law of molds. The identified molds include *Aspergillus niger*, *Penicillium rubens*, *Rhizopus microsporus*, *Penicillium oxalicum*, and *Aspergillus versicolor*. Utilizing GC‐IMS technology, we characterized the mVOCs associated with these dominant molds and the VOCs present in moldy soybeans. A total of 22 characteristic mVOCs were identified from the dominant molds, while 70 VOCs were detected in moldy soybeans. As the mildew degree increased, the concentrations of nonanal, octanal, decanal, heptanal, and valeraldehyde progressively decreased, contrasting with the rising levels of phenylacetaldehyde, 3‐methyl‐2‐butenal, 3‐methylbutyraldehyde, 3‐methylthiopropionaldehyde, and 3‐octanone. These characteristic VOCs, identified through the analysis of dominant mold mVOCs, serve as indicators for assessing soybean mildew. PCA and clustering results revealed significant differences in VOCs across different soybean mildew degrees, indicating changes in content with the advancement of mildew. Therefore, in conclusion, the integration of GC‐IMS with chemometrics effectively distinguishes soybean samples with varying mildew degrees. This approach provides valuable data for developing mildew detection methods and ensuring quality and safety measures.

## AUTHOR CONTRIBUTIONS

**Xuejian Song:** Data curation (equal); investigation (equal); supervision (equal); validation (equal); writing – original draft (equal); writing – review and editing (equal). **Lili Qian:** Data curation (equal); funding acquisition (equal); project administration (equal); writing – review and editing (equal). **Lixue Fu:** Investigation (equal); methodology (equal); writing – original draft (equal). **Rongan Cao:** Software (equal); writing – review and editing (equal). **Xinhui Wang:** Investigation (equal); methodology (equal); software (equal). **Mingming Chen:** Formal analysis (equal); investigation (equal); methodology (equal).

## FUNDING INFORMATION

The National Key Research and Development Program of China. “Grain transportation from north to south” integrated research and development of key technologies and equipment for anti‐condensation and anti‐mildew in container transportation of bulk grain (grant number: 2018YFD0401403). Coarse cereals Industry Technology Collaborative Innovation System Coarse cereals Quality Traceability Technology Post and Characteristic Discipline Funding Project from Heilongjiang Province, China (Hei Education Union [2018] No. 4).

## CONFLICT OF INTEREST STATEMENT

The authors have no conflict of interest to declare.

## CONSENT FOR PUBLICATION

The authors hereby consent to the publication of the work.

## Data Availability

The data that support the findings of this study are available on request from the corresponding author.

## References

[fsn34302-bib-0001] Baek, J. , Lee, E. , Kim, N. , Kim, S. L. , Choi, I. , Ji, H. , & Kim, K.‐H. (2020). High throughput phenotyping for various traits on soybean seeds using image analysis. Sensors, 20(1), 248. 10.3390/s20010248 31906262 PMC6982885

[fsn34302-bib-0002] Brodsky, M. H. , Entis, P. , Entis, M. P. , Sharpe, A. N. , & Jarvis, G. A. (1982). Determination of aerobic plate and yeast and mold counts in foods using an automated hydrophobic grid‐membrane filter technique. Journal of Food Protection, 45(4), 301–304. 10.4315/0362-028X-45.4.301 30866328

[fsn34302-bib-0003] Capitain, C. , & Weller, P. (2021). Non‐targeted screening approaches for profiling of volatile organic compounds based on gas chromatography‐ion mobility spectroscopy (GC‐IMS) and machine learning. Molecules, 26(18), 5457. 10.3390/molecules26185457 34576928 PMC8468721

[fsn34302-bib-0004] Chelladurai, V. , Jayas, D. S. , & White, N. D. G. (2010). Thermal imaging for detecting fungal infection in stored wheat. Journal of Stored Products Research, 46(3), 174–179. 10.1016/j.jspr.2010.04.002

[fsn34302-bib-0005] Chen, Y. , Xu, H. , Ding, S. , Zhou, H. , Qin, D. , Deng, F. , & Wang, R. (2020). Changes in volatile compounds of fermented minced pepper during natural and inoculated fermentation process based on headspace–gas chromatography–ion mobility spectrometry. Food Science & Nutrition, 8(7), 3362–3379. 10.1002/fsn3.1616 32724601 PMC7382115

[fsn34302-bib-0006] National Standard of the People's Republic of China . (2016). Food microbiological examination mould and yeast count, GB 4789.15–2016 .

[fsn34302-bib-0007] Fox, G. , & Manley, M. (2014). Applications of single kernel conventional and hyperspectral imaging near infrared spectroscopy in cereals. Journal of the Science of Food and Agriculture, 94(2), 174–179. 10.1002/jsfa.6367 24038031

[fsn34302-bib-0008] Gallegos, J. , Arce, C. , Jordano, R. , Arce, L. , & Medina, L. M. (2017). Target identification of volatile metabolites to allow the differentiation of lactic acid bacteria by gas chromatography‐ion mobility spectrometry. Food Chemistry, 220, 362–370. 10.1016/j.foodchem.2016.10.022 27855912

[fsn34302-bib-0009] García‐Nicolás, M. , Arroyo‐Manzanares, N. , Arce, L. , Hernández‐Córdoba, M. , & Viñas, P. (2020). Headspace gas chromatography coupled to mass spectrometry and ion mobility spectrometry: Classification of virgin olive oils as a study case. Food, 9(9), 1288. 10.3390/foods9091288 PMC7555980 32937810

[fsn34302-bib-0010] Gonzalez Pereyra, M. L. , Rosa, C. A. R. , Dalcero, A. M. , & Cavaglieri, L. R. (2011). Mycobiota and mycotoxins in malted barley and brewer's spent grain from Argentinean breweries. Letters in Applied Microbiology, 53(6), 649–655. 10.1111/j.1472-765X.2011.03157.x 21967240

[fsn34302-bib-0011] Gu, S. , Chen, W. , Wang, Z. , & Wang, J. (2021). Rapid determination of potential aflatoxigenic fungi contamination on peanut kernels during storage by data fusion of HS‐GC‐IMS and fluorescence spectroscopy. Postharvest Biology and Technology, 171, 111361. 10.1016/j.postharvbio.2020.111361

[fsn34302-bib-0012] Gu, S. , Chen, W. , Wang, Z. , Wang, J. , & Huo, Y. (2020). Rapid detection of Aspergillus spp. infection levels on milled rice by headspace‐gas chromatography ion‐mobility spectrometry (HS‐GC‐IMS) and E‐nose. LWT, 132, 109758. 10.1016/j.lwt.2020.109758

[fsn34302-bib-0013] Gu, S. , Wang, Z. , Chen, W. , & Wang, J. (2020). Targeted versus nontargeted green strategies based on headspace‐gas chromatography–ion mobility spectrometry combined with chemometrics for rapid detection of fungal contamination on wheat kernels. Journal of Agricultural and Food Chemistry, 68(45), 12719–12728. 10.1021/acs.jafc.0c05393 33124819

[fsn34302-bib-0014] Gu, S. , Wang, Z. , & Wang, J. (2021). Untargeted rapid differentiation and targeted growth tracking of fungal contamination in rice grains based on headspace‐gas chromatography‐ion mobility spectrometry. Journal of the Science of Food and Agriculture, 102(9), 3673–3682. 10.1002/jsfa.11714 34890123

[fsn34302-bib-0015] Han, Y. , Wang, C. , Zhang, X. , Li, X. , & Gao, Y. (2022). Characteristic volatiles analysis of Dongbei Suancai across different fermentation stages based on HS‐GC‐IMS with PCA. Journal of Food Science, 87(2), 612–622. 10.1111/1750-3841.16045 35067929

[fsn34302-bib-0016] He, J. , Ye, L. , Li, J. , Huang, W. , Huo, Y. , Gao, J. , Liu, L. , & Zhang, W. (2021). Identification of Ophiopogonis Radix from different producing areas by headspace‐gas chromatography‐ion mobility spectrometry analysis. Journal of Food Biochemistry, 46(6), e13850. 10.1111/jfbc.13850 34227128

[fsn34302-bib-0017] Hidaka, Y. , & Kubota, K. (2006). Study on the sterilization of grain surface using UV radiation: Development and evaluation of UV irradiation equipment. Jarq‐Japan Agricultural Research Quarterly, 40, 157–161. 10.6090/JARQ.40.157

[fsn34302-bib-0018] Grain Industry Standard of the People's Republic of China . (2018). *Inspection of grain and oils—Storage fungal examination‐Enumeration spores of fungi*. LS/T6132‐2018.

[fsn34302-bib-0019] Li, Q. , Zuo, Y. , Wang, X. , Jiang, S. , Wang, S. , & Hou, L. (2024). Sensitivity analysis of container properties to heating uniformity of soybeans during radio frequency treatment. Journal of Food Engineering, 365, 111822. 10.1016/j.jfoodeng.2023.111822

[fsn34302-bib-0020] Liu, J. , Deng, J.‐C. , Yang, C.‐Q. , Huang, N. , Chang, X.‐L. , Zhang, J. , & Yang, W.‐Y. (2017). Fungal diversity in field mold‐damaged soybean fruits and pathogenicity identification based on high‐throughput rDNA sequencing. Frontiers in Microbiology, 8, 779. 10.3389/fmicb.2017.00779 28515718 PMC5413577

[fsn34302-bib-0021] Liu, X. , Bai, Y. , & Chen, J. (2017). An intermodal transportation geospatial network modeling for containerized soybean shipping. Journal of Ocean Engineering and Science, 2(2), 143–153. 10.1016/j.joes.2017.05.001

[fsn34302-bib-0022] Lv, W. , Ye, L. , & Wang, L. (2022). Changes of China's soybean import market power and influencing factors. Applied Economics Letters, 30(18), 2619–2625. 10.1080/13504851.2022.2101604

[fsn34302-bib-0023] Ma, N. , Guan, R. , Zhao, R. , Geng, Y. , & Picone, G. (2022). GC‐IMS‐based preliminary analysis of volatile flavor compounds in Ejiao at different processing stages. Journal of Food Quality, 2022, 1–12. 10.1155/2022/3961593

[fsn34302-bib-0024] Mahlein, A.‐K. , Alisaac, E. , Al Masri, A. , Behmann, J. , Dehne, H.‐W. , & Oerke, E.‐C. (2019). Comparison and combination of thermal, fluorescence, and hyperspectral imaging for monitoring fusarium head blight of wheat on spikelet scale. Sensors, 19(10), 2281. 10.3390/s19102281 31108868 PMC6567885

[fsn34302-bib-0025] Mateo, E. M. , Gil‐Serna, J. , Patiño, B. , & Jiménez, M. (2011). Aflatoxins and ochratoxin A in stored barley grain in Spain and impact of PCR‐based strategies to assess the occurrence of aflatoxigenic and ochratoxigenic Aspergillus spp. International Journal of Food Microbiology, 149(2), 118–126. 10.1016/j.ijfoodmicro.2011.06.006 21741104

[fsn34302-bib-0026] Meng, X. (2020). The analysis of the motivation and conditions of the “scattered change set” Cargo. IOP Conference Series: Earth and Environmental Science, 546(3), 032038. 10.1088/1755-1315/546/3/032038

[fsn34302-bib-0027] Pan, L. , Farouk, M. , Qin, G. , Zhao, Y. , & Bao, N. (2018). The influences of soybean agglutinin and functional oligosaccharides on the intestinal tract of monogastric animals. International Journal of Molecular Sciences, 19(2), 554. 10.3390/ijms19020554 29439523 PMC5855776

[fsn34302-bib-0028] Rizzo, G. , & Baroni, L. (2018). Soy, soy foods and their role in vegetarian diets. Nutrients, 10(1), 43. 10.3390/nu10010043 29304010 PMC5793271

[fsn34302-bib-0029] Shen, F. , Wu, Q. , Liu, P. , Jiang, X. , Fang, Y. , & Cao, C. (2018). Detection of Aspergillus spp. contamination levels in peanuts by near infrared spectroscopy and electronic nose. Food Control, 93, 1–8. 10.1016/j.foodcont.2018.05.039

[fsn34302-bib-0030] Singh, S. K. , Barman, M. , Prasad, J. P. , & Bahuguna, R. N. (2022). Phenotyping diverse wheat genotypes under terminal heat stress reveal canopy temperature as critical determinant of grain yield. Plant Physiology Reports, 27(2), 335–344. 10.1007/s40502-022-00647-y

[fsn34302-bib-0031] Speckbacher, V. , Zeilinger, S. , Zimmermann, S. , Mayhew, C. A. , Wiesenhofer, H. , & Ruzsanyi, V. (2021). Monitoring the volatile language of fungi using gas chromatography‐ion mobility spectrometry. Analytical and Bioanalytical Chemistry, 413(11), 3055–3067. 10.1007/s00216-021-03242-6 33675374 PMC8043876

[fsn34302-bib-0032] Thomas, C. F. , Zeh, E. , Dörfel, S. , Zhang, Y. , & Hinrichs, J. (2021). Studying dynamic aroma release by headspace‐solid phase microextraction‐gas chromatography‐ion mobility spectrometry (HS‐SPME‐GC‐IMS): Method optimization, validation, and application. Analytical and Bioanalytical Chemistry, 413(9), 2577–2586. 10.1007/s00216-021-03222-w 33655348

[fsn34302-bib-0033] Vautz, W. , Franzke, J. , Zampolli, S. , Elmi, I. , & Liedtke, S. (2018). On the potential of ion mobility spectrometry coupled to GC pre‐separation – A tutorial. Analytica Chimica Acta, 1024, 52–64. 10.1016/j.aca.2018.02.052 29776547

[fsn34302-bib-0034] Yang, Y. , Fang, B. , Feng, S. , Wang, Z. , Luo, Z. , Yao, Z. , Zou, H. , & Huang, L. (2021). Isolation and identification of *Trichoderma asperellum*, the novel causal agent of green mold disease in Sweetpotato. Plant Disease, 105(6), 1711–1718. 10.1094/pdis-07-20-1484-re 33373292

[fsn34302-bib-0035] Yang, Y. , Wang, B. , Fu, Y. , Shi, Y.‐G. , Chen, F.‐L. , Guan, H.‐N. , & Zhang, N. (2021). HS‐GC‐IMS with PCA to analyze volatile flavor compounds across different production stages of fermented soybean whey tofu. Food Chemistry, 346, 128880. 10.1016/j.foodchem.2020.128880 33418415

[fsn34302-bib-0036] Yu, F.‐Y. , Vdovenko, M. M. , Wang, J.‐J. , & Sakharov, I. Y. (2011). Comparison of enzyme‐linked immunosorbent assays with chemiluminescent and colorimetric detection for the determination of ochratoxin a in food. Journal of Agricultural and Food Chemistry, 59(3), 809–813. 10.1021/jf103261u 21204536

[fsn34302-bib-0037] Zareef, M. , Arslan, M. , Hassan, M. M. , Ahmad, W. , Ali, S. , Li, H. , & Chen, Q. (2021). Recent advances in assessing qualitative and quantitative aspects of cereals using nondestructive techniques: A review. Trends in Food Science & Technology, 116, 815–828. 10.1016/j.tifs.2021.08.012

[fsn34302-bib-0038] Zhang, G. , Li, P. , Zhang, W. , & Zhao, J. (2017). Analysis of multiple soybean phytonutrients by near‐infrared reflectance spectroscopy. Analytical and Bioanalytical Chemistry, 409(14), 3515–3525. 10.1007/s00216-017-0288-8 28424855

[fsn34302-bib-0039] Zhang, S. , Hao, D. , Zhang, S. , Zhang, D. , Wang, H. , Du, H. , & Yu, D. (2020). Genome‐wide association mapping for protein, oil and water‐soluble protein contents in soybean. Molecular Genetics and Genomics, 296(1), 91–102. 10.1007/s00438-020-01704-7 33006666

[fsn34302-bib-0040] Zhu, Y.‐L. , Zhang, H.‐S. , Zhao, X.‐S. , Xue, H.‐H. , Xue, J. , & Sun, Y.‐H. (2018). Composition, distribution, and antioxidant activity of phenolic compounds in 18 soybean cultivars. Journal of AOAC International, 101(2), 520–528. 10.5740/jaoacint.17-0156 28847347

